# Hemolytic Anemia Linked to Epstein–Barr Virus Infectious Mononucleosis: A Systematic Review of the Literature

**DOI:** 10.3390/jcm14041283

**Published:** 2025-02-15

**Authors:** Dario F. Meloni, Pietro B. Faré, Gregorio P. Milani, Sebastiano A. G. Lava, Mario G. Bianchetti, Samuele Renzi, Massimiliano Bertacchi, Lisa Kottanattu, Gabriel Bronz, Pietro Camozzi

**Affiliations:** 1 Family Medicine, Faculty of Biomedical Sciences, Università Della Svizzera Italiana, 6900 Lugano, Switzerlandpietro.fare@eoc.ch (P.B.F.); gabriel.bronz@eoc.ch (G.B.); 2 Department of Infectious Diseases and Hospital Epidemiology, University Hospital Zurich, 8091 Zurich, Switzerland; 3 Pediatric Unit, Fondazione IRCCS Ca’ Granda Ospedale Maggiore Policlinico, 20122 Milan, Italy; gregorio.milani@unimi.it; 4 Department of Clinical Sciences and Community Health, Università Degli Studi di Milano, 20122 Milan, Italy; 5 Pediatric Cardiology Unit, Department of Pediatrics, Centre Hospitalier Universitaire Vaudois and University of Lausanne, 1011 Lausanne, Switzerland; sebastiano.lava@chuv.ch; 6 Division of Hematology and Oncology, CHUL-Laval, Quebec City, QC G1V 0A6, Canada; samuele.renzi.med@ssss.gouv.qc.ca; 7 Department of Pediatrics, Laval University, Quebec City, QC G1V 4G2, Canada; 8 Pediatric Nephrology Unit, Geneva University Hospitals, 1206 Geneva, Switzerland; massimiliano.bertacchi@huge.ch; 9 Pediatric Institute of Southern Switzerland, Ente Ospedaliero Cantonale, 6900 Bellinzona, Switzerland; lisa.kottanattu@eoc.ch; 10 Faculty of Biomedical Sciences, Università Della Svizzera Italiana, 6900 Lugano, Switzerland; 11 Department of Internal Medicine, Regional Hospital of Locarno, Ente Ospedaliero Cantonale, 6900 Locarno, Switzerland; 12 Department of Anesthesia, Hôpital du Valais, 1950 Sion, Switzerland; pietro.camozzi@hopitalvs.ch

**Keywords:** Epstein–Barr virus, infectious mononucleosis, hemolysis, cold antibodies, warm antibodies

## Abstract

**Background**: In Epstein–Barr virus infectious mononucleosis, hemolytic anemia occasionally occurs. **Methods**: To characterize hemolytic anemia linked to Epstein–Barr virus infectious mononucleosis, we performed a systematic review (PROSPERO CRD42024597183) in the United States National Library of Medicine, Excerpta Medica, and Web of Science with no restrictions on language. Only reports published since 1970 were included. Eligible were reports describing hemolytic anemia in subjects with clinical signs and microbiological markers of Epstein–Barr virus mononucleosis. **Results**: In the literature, we detected 56 reports released between 1973 and 2024, documenting 60 individuals (32 females and 28 males; 27 children and 33 adults) with hemolytic anemia linked to Epstein–Barr virus infectious mononucleosis. The mechanism underlying anemia was categorized as cold-antibody-mediated (N = 31; 52%), warm-antibody-mediated (N = 18, 30%), mixed warm- and cold-antibody-mediated (N = 4; 6.7%), or paroxysmal cold hemoglobinuria (N = 2; 3.3%). The remaining 5 cases (8.3%) remained unclassified. Observation alone was the chosen approach in 23% of cases (N = 14). Steroids (67%; N = 40) and blood transfusions (38%; N = 23) were the most commonly used treatment, while plasma exchange, intravenous polyclonal immunoglobulin, rituximab, and splenectomy were used less frequently. Observation was slightly but significantly (*p* = 0.032) more common in cases of cold-antibody-mediated anemia compared to all other cases combined. Patients recovered a median of 28 [interquartile range 21–39] days after disease onset. Two patients with warm-antibody-mediated hemolytic anemia died. **Conclusions**: This literature review points out that Epstein–Barr virus, like Mycoplasma pneumoniae, cytomegalovirus, or severe acute respiratory syndrome coronavirus 2, may act as a trigger for immune-mediated hemolytic anemia.

## 1. Introduction

Epstein–Barr virus typically causes infectious mononucleosis, also known as glandular fever. This infection predominantly affects individuals aged 15 to 25 and manifests with malaise, fatigue, fever, sore throat, hepatomegaly, splenomegaly, lymphadenopathy, and periorbital swelling. In most cases, these features resolve spontaneously within one month [1,2]. The typical Epstein–Barr virus glandular fever is very common in high-income countries, whereas it is relatively rare (or even very rare) in low- and middle-income countries [1,2].

Acute hemolysis may occur in Epstein–Barr virus infectious mononucleosis. To date, this complication, first reported in 1943 [3], has been documented uniquely in individual case reports or in very small case series. Although case reports offer valuable insights into rare conditions, only their collective analysis through systematic reviews can reveal consistent patterns, clarify clinical characteristics, and uncover treatment strategies that may remain hidden in isolated cases. Such reviews not only enhance patient care and inform clinical decisions but also identify critical areas for future research, driving progress in the field.

To further explore acute hemolysis linked with Epstein–Barr virus infectious mononucleosis, we carried out a systematic review of the literature.

## 2. Materials and Methods

### 2.1. Study Preregistration–Date Sources–Search Strategy

The systematic review was prospectively registered [4] with the International Prospective Register of Systematic Reviews (CRD42024597183) and was conducted in line with the Preferred Reporting Items for Systematic Reviews and Meta-Analyses guidelines [5]. The databases used for this investigation included the United States National Library of Medicine, Excerpta Medica, and Web of Science with no restrictions on language. Only reports published since 1970 were included.

The search methodology employed the following combinations of keywords: (Drüsenfieber OR Epstein–Barr virus OR glandular fever OR herpesvirus type 4 OR infectious mononucleosis) AND (Coombs test OR direct antiglobulin test OR hemolysis OR hemolytic anemia). Additionally, articles cited in the reference lists of retrieved records, and articles already known to the authors were also considered [6]. The searches were conducted in October 2024 and repeated just once prior to submission on 20 December 2024.

### 2.2. Definitions

The clinical diagnosis of infectious mononucleosis was made in individuals without a history of immunodeficiency, autoimmunity, or previous mononucleosis, who exhibited at least three of the following seven features: malaise or fatigue, fever, sore throat, hepatomegaly, splenomegaly, lymphadenopathy, and eyelid swelling [1,2]. The diagnosis was also accepted if the term “clinical mononucleosis” was used by the report’s authors, even without a description of symptoms and signs.

The microbiological diagnosis of Epstein–Barr virus mononucleosis was based on positive serological findings, including a positive Paul-Bunnell-Davidsohn heterophile antibody test, the presence of immunoglobulin M (with or without immunoglobulin G) against the viral capsid antigen, or immunoglobulin G against the early viral antigen, and was also established when the viral genome was detected in the blood [1,2]. Individuals with only clinical signs or microbiological markers of Epstein–Barr virus mononucleosis were excluded.

The diagnosis of acute hemolysis [7] was made in patients presenting with sudden-onset anemia, accompanied by markers of increased red blood cell destruction (elevated lactate dehydrogenase or low haptoglobin) or production (polychromasia or reticulocytosis). Individuals without a direct Coombs antiglobulin test were excluded. A positive result for this test was deemed essential only in cases with a pre-existing congenital disease prone to hemolysis. Yellowish eye or skin discoloration and hyperbilirubinemia were not included in the criteria for assessing increased red blood cell destruction because these features may be common also in acute liver disease associated with infectious mononucleosis [1,2]. The chronological relationship to mononucleosis was used to classify anemia as intra-infectious if it occurred before resolution of mononucleosis, or as post-infectious if it arose within 14 days afterwards. Hemolytic anemia was categorized into cold-antibody-mediated, warm-antibody-mediated, mixed warm- and cold-antibody-mediated, paroxysmal cold hemoglobinuria, or unclassified, as recommended in the literature [7,8]. Cases presenting with a hemolytic-uremic syndrome temporally associated with an infectious mononucleosis were excluded [9].

Additional definitions [10,11] employed for the current analysis are provided in Table 1.

### 2.3. Data Extraction

Five data sets were extracted for each individual experiencing acute hemolysis temporally associated with Epstein–Barr virus: 1. demographics, pre-existing congenital disease prone to hemolysis; 2. clinical data including further complications other than hemolytic anemia; 3. laboratory data supporting the diagnosis of acute Epstein–Barr virus infectious mononucleosis; 4. results of tests conducted to identify the mechanisms behind hemolytic anemia; 5. management and disease course. The data were extracted using a piloted form and then transcribed into a predefined spreadsheet.

### 2.4. Reporting Completeness–Statistical Analysis

For each individual case, the completeness of reporting for the five afore-mentioned data sets was evaluated on a scale of 0, 1, or 2. Based on the total score, the overall reporting comprehensiveness for each case was then rated as excellent (9 or 10), good (7 or 8), or satisfactory (5 to 7), according to our standard procedure [12]. Two authors independently and in duplicate conducted the literature search, selected the reports for inclusion, gathered the data, and assessed the comprehensiveness of each reported case. Disagreements were addressed through discussion, with a senior author consulted if necessary. One author input the data into the spreadsheet, and another cross-checked it for accuracy.

Pairwise deletion was utilized to handle missing data. Categorical data are presented as counts (and sometimes as percentages) and were analyzed using the Fisher test. Percentages were approximated to the nearest whole number for values of 10 or higher and to one decimal place for values under 10. Continuous variables are shown as medians with interquartile ranges and were evaluated using the non-parametric Kruskal-Wallis H test [13]. A two-sided *p*-value threshold of less than 0.05 was used to evaluate statistical significance. Analyses were carried out using GraphPad Prism version 10.4.1 (GraphPad Software, San Diego, CA, USA).

## 3. Results

### 3.1. Search Results

**Figure 1:**
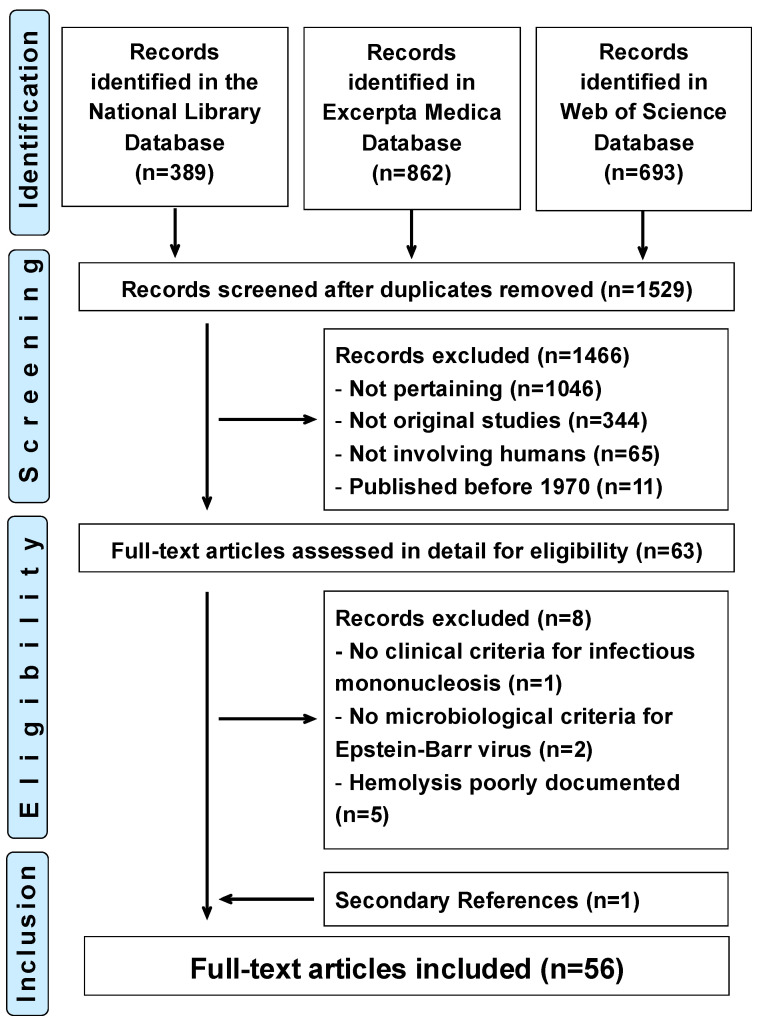
Hemolytic anemia linked to Epstein–Barr virus infectious mononucleosis. Flowchart of the literature search. No new pertinent reports were identified in the literature search updated on 20 December 2024.

The study flowchart is presented in Figure 1.

Fifty-six reports [14,15,16,17,18,19,20,21,22,23,24,25,26,27,28,29,30,31,32,33,34,35,36,37,38,39,40,41,42,43,44,45,46,47,48,49,50,51,52,53,54,55,56,57,58,59,60,61,62,63,64,65,66,67,68,69] released between 1973 and 2024 were included in the final analysis, documenting cases of hemolytic anemia in individuals with acute Epstein–Barr virus infectious mononucleosis. The articles were written in English (N = 50), Spanish (N = 3), French (N = 1), German (N = 1), and Italian (N = 1). They came from the following continents: 28 from Europe (Spain, N = 6; the United Kingdom, N = 5; Türkiye, N = 4; Italy, N = 3; Croatia, N = 2; France, N = 2; Greece, N = 2; Germany, N = 1; Portugal, N = 1; Sweden, N = 1, Switzerland, N = 1), 19 from America (United States, N = 15; Canada, N = 3, Brazil N = 1), 6 from Asia (India, N = 3; Sri Lanka, N = 2; Israel, N = 1), 2 from Oceania (Australia), and 1 from Africa (South Africa).

The 56 selected articles [14,15,16,17,18,19,20,21,22,23,24,25,26,27,28,29,30,31,32,33,34,35,36,37,38,39,40,41,42,43,44,45,46,47,48,49,50,51,52,53,54,55,56,57,58,59,60,61,62,63,64,65,66,67,68,69] reported on 60 individuals with acute hemolytic anemia temporally associated with Epstein–Barr virus infectious mononucleosis. Reporting completeness was excellent in 13 (22%), good in 38 (63%), and satisfactory in 9 (15%) cases.

### 3.2. Findings

The following microbiological studies were used to support the clinical diagnosis of Epstein–Barr virus infectious mononucleosis in the 60 patients (two tests were used in 24 and one in 36 patients): Paul-Bunnell-Davidsohn heterophile antibody test, N = 32; detection of immunoglobulin M (with or without immunoglobulin G) against the Epstein–Barr viral capsid antigen, N = 36; detection of immunoglobulin G against the early Epstein–Barr viral antigen, N = 9; detection of Epstein–Barr virus genome in blood, N = 7.

The characteristics of the 60 patients, comprising 27 children and 33 adults with an almost equal distribution between males and females, are presented in Table 2.

Hemolytic anemia was nearly always intra-infectious. Two patients had a pre-existing condition predisposing to hemolysis. Cytopenias, aside from hemolytic anemia, were observed in just over one-fifth of the cases. Acute liver disease (62%) and neurological disease (6.7%) were the most frequently reported non-hematological complications.

The mechanism underlying anemia was categorized as cold-antibody-mediated (N = 31; 52%), warm-antibody-mediated (N = 18, 30%), mixed warm- and cold-antibody-mediated (N = 4; 6.7%), or paroxysmal cold hemoglobinuria (N = 2; 3.3%). The remaining 5 cases (8.3%) remained unclassified. Female-to-male ratio and age were similar among patients with cold-antibody-mediated anemia, with warm-antibody-mediated anemia and in the remaining 11 cases (Table 3).

Hemolytic anemia was temporally associated with an otherwise unexplained acute kidney injury in 10% of cases. None of the patients with an acute kidney injury underwent a diagnostic kidney biopsy.

Observation alone was the chosen approach in 23% of cases. Steroids (67%) and blood transfusions (38%) were the most commonly used treatments, while plasma exchange, intravenous polyclonal immunoglobulin, rituximab, and splenectomy were used less frequently. Observation was slightly but significantly (*p* = 0.032) more common in cases of cold-antibody-mediated anemia compared to all other cases combined.

### 3.3. Course–Outcome

Patients recovered a median of 28 [21–39] days after disease onset, with disease duration of ≥60 days observed in slightly less than 10% of cases. Two patients with warm-antibody-mediated hemolytic anemia died: a 5-month-old female infant, who was concurrently affected by severe encephalitis and hepatitis [40], and a 31-year-old man, whose course was complicated by a severe systemic bacterial infection [50]. Disease duration was similar (*p* = 0.8555) in 13 cases with (29 [20–40] days) and 47 cases without (31 [22–39] days) other cytopenias.

## 4. Discussion

This systematic literature review indicates that Epstein–Barr virus infectious mononucleosis chronologically associated with hemolysis affects individuals of both sexes, with an age distribution similar to that of those affected by uncomplicated mononucleosis [1,2]. The hemolytic anemia linked to Epstein–Barr virus infectious mononucleosis develops during the course of infectious mononucleosis, is frequently accompanied by thrombocytopenia or leukopenia, and sometimes by acute kidney injury. Finally, this form of hemolytic anemia can be fatal in the presence of further severe Epstein–Barr virus mononucleosis complications, confirming that, in infectious mononucleosis, death is typically due to complications such as splenic rupture, airway obstruction, neurological involvement, or renal dysfunction, rather than the disease itself [1,2].

The mechanisms underlying hemolytic anemia linked to Epstein–Barr virus infectious mononucleosis seem to be immune-mediated [7,8]. In over 95% of cases where the mechanism has been identified, warm antibodies, cold antibodies, or both are detected, with cold antibodies being the most frequent. Cold antibodies are generally monoclonal or oligoclonal immunoglobulins of class M that bind to red cells at temperatures below normal body temperature. However, in hemolytic anemia associated with Epstein–Barr virus infectious mononucleosis, cold antibodies are polyclonal [8,70]. Warm antibodies are predominantly polyclonal immunoglobulins, primarily of class G, that react to red cells at normal body temperature [7,71].

This analysis is based on data collected over 50 years and, as such, cannot guide therapy but suggests that hemolytic anemia from cold antibodies may more often be managed with observation alone compared to hemolytic anemia from warm antibodies.

Nowadays, it is strongly advised to carefully tailor the treatment of immune-mediated hemolytic anemia to the underlying pathophysiological mechanism [7,8,70,71,72]. Cold antibody-mediated cases triggered by Epstein–Barr virus mononucleosis (cold-agglutinin syndrome) typically resolve spontaneously. As cold antibodies bind to red cells in cooler areas (e.g., extremities), thermal protection is the cornerstone of therapy. Steroids have limited, if any, documented efficacy, and the efficacy of complement inhibitors remains unproven. In warm-antibody mediated cases, steroids are first-line therapy, with rituximab as the recommended second-line treatment. Immunosuppressants and splenectomy have been second-line options for the past 20–40 years. Both in cold- and warm-antibody-mediated hemolysis, blood transfusion should be reserved for severe cases, requiring specific preparation and a dedicated team.

Intravascular hemolysis releases free hemoglobin, which can harm the kidneys and cause acute kidney injury, as seen in 10% of the cases included in this review [73]. Interstitial nephritis directly linked to the Epstein–Barr virus, myositis and drug induced kidney damage are further possible causes of acute kidney injury in this setting [74,75].

The merits of this literature review include the pre-registration [4], a rigorous case selection methodology [5], and a broad temporal scope spanning 50 years. However, limitations include the incomplete reporting of some cases, the challenge of not knowing the prevalence of this condition, and the lack of robust evidence to establish therapeutic recommendations. This study highlights the necessity of tailoring treatment to the specific immune-mediated mechanism and paves the way for future research into more effective therapies.

Epstein–Barr virus also causes infections in infancy and childhood, particularly in middle- and low-income countries. In this age group, the presentation differs from classic Epstein–Barr virus glandular fever and is characterized by nonspecific features, including otitis, cough, rhinitis, diarrhea, and abdominal complaints [1,2]. The present literature review included only subjects presenting with both the clinical features of infectious mononucleosis and markers of Epstein–Barr virus infection. Consequently, cases of Epstein–Barr virus infections affecting infants and children were not identified by our literature search.

## 5. Conclusions

The earliest descriptions of infectious mononucleosis are usually credited to two pediatricians: F. N. F. Filatov from Russia in 1885 and E. Pfeiffer from Germany in 1889. The latter described the illness as “Drüsenfieber” (glandular fever). In 1920, T.P. Sprunt and F.A. Evans observed an increase in mononuclear cells in the blood and coined the term infectious mononucleosis. A significant milestone followed in 1923 when H. Downey and C.A. McKinlay provided a detailed description of the atypical lymphocyte. The disease became a distinct clinical entity in the 1930s, thanks to J.R. Paul and W.W. Bunnell, who, along with J. Davidsohn and P.H. Walker, developed the heterophile antibody test for diagnosis, which is often referred to as the Paul-Bunnell(-Davidsohn) test. Finally, in the 1960s and 1970s, the Epstein–Barr virus, now often known as human herpesvirus 4, was identified as the causative agent of infectious mononucleosis [1,2].

The findings of this systematic literature review point out that Epstein–Barr virus, like Mycoplasma pneumoniae, cytomegalovirus, or severe acute respiratory syndrome coronavirus 2, may act as a trigger for immune-mediated hemolytic anemia [3,76,77,78,79].

## Figures and Tables

**Table 1:** Additional definitions utilized for the present analysis.

Further cytopenias [1,2] Trombocytopenia: ≤149 × 109 cells/L - Mild: 101–149 × 109 cells/L - Moderate: 21–100 × 109 cells/L - Severe: ≤20 × 109 cells/L Leukopenia: ≤4.4 × 109 cells/L Acute liver disease: alanine aminotransferase, aspartate aminotransferase, alkaline phosphatase, ɣ-glutamyltransferase, or leucine aminopeptidase at least two times the upper limit of normal [1]. Myositis: muscle weakness, pain or swelling, total creatine kinase level at least five times the upper limit of normal, and absent signs consistent with an ischemic myocardial disease [10]. Acute kidney injury [11]: (a) increase in blood creatinine of ≥27 µmol/L or ≥50% within 48 h; (b) categorization based on creatinine level: stage I (increase of 1.5 to 1.9 times the baseline), stage II (increase of 2.0 to 2.9 times the baseline), stage III (creatinine increase of ≥3.0 times the baseline, increase of ≥354 μmol/L, or initiation of kidney replacement therapy).

**Table 2:** Characteristics of 60 patients 5 months to 70 years of age with acute hemolysis temporally associated with Epstein–Barr virus infectious mononucleosis. Results are presented either as value (and sometimes also as percentage) or as median [and interquartile range].

N	60
**Demographics**	
Females:males	32 (53):28 (47)
Age	
years	20 [16–25]
≤18 years	27 (45)
**Co-existing inherited anemia**	
Elliptocytosis	1
Thalassemia	1
**Chronological relationship to mononucleosis**	
Intra-infectious	59 (98)
Post-infectious	1 (1.7)
**Cytopenias other than anemia**	13
Thrombocytopenia	11
mild	6
moderate	4
severe	1
Leukopenia	2
**Further complications**	
Acute liver disease	37
Neurological complications	4
Encephalitis	1
Guillain–Barré syndrome	1
Transverse myelitis	1
Unilateral sensorineural deafness	1
Pancreatitis	2
Nephrotic syndrome	1
Synovitis	1
Myositis	0

**Table 3:** Demographics, prevalence of acute kidney injury, management and outcome of 60 patients 5 months to 70 years of age with acute hemolysis temporally associated with Epstein–Barr virus infectious mononucleosis based on the categorization of anemia. Results are presented either as value (and sometimes also as percentage) or as median [and interquartile range].

	All	Cold-Antibody Mediated	Warm-Antibody Mediated	Remaining	*p*-Value
**N**	60	31	18	11	
**Demographics**					
Females:Males	32:28	18:13	9:9	5:6	0.7858
Age, years	20 [16–25]	20 [16–26]	23 [6.5–37]	18 [16–21]	0.4298
**Acute kidney injury**	6	2	4	0	0.1716
Stage I	1	0	1	0	
Stage II	1	0	1	0	
Stage III	4	2	2	0	
**Management**					
Exclusively expectant	14 (23)	11 (35)	2 (11)	1 (9.0)	0.0320 *
Immunomodulation	40 (67)	16 (52)	16 (89)	8 (73)	0.0233 *
Steroids	40	16	16	8	
Plasma exchange	5	2	2	1	
Polyclonal immunoglobulin	4	0	3	1	
Rituximab	3	1	2	0	
Splenectomy	1	0	1	0	
Blood cell transfusions	23 (38)	9 (29)	8 (44)	6 (55)	0.2745
**Disease duration**					
Days	28 [21–39]	28 [21–39]	26 [16–38]	31 [21–40]	0.4309
≥60 days	4	2	1	1	>0.9999
**Lethal course**	2	0	2	0	0.1175

* Cold-antibody mediated versus warm-antibody mediated and remaining.

## Data Availability

Data sharing is not applicable to this report as no new data were produced during this study.
